# Acute Changes in Carotid-Femoral Pulse-Wave Velocity Are Tracked by Heart-Femoral Pulse-Wave Velocity

**DOI:** 10.3389/fcvm.2020.592834

**Published:** 2021-01-20

**Authors:** Keeron Stone, Simon Fryer, James Faulkner, Michelle L. Meyer, Gabriel Zieff, Craig Paterson, Kathryn Burnet, Elizabeth Kelsch, Daniel Credeur, Danielle Lambrick, Lee Stoner

**Affiliations:** ^1^School of Sport and Exercise, University of Gloucestershire, Gloucester, United Kingdom; ^2^Department of Sport, Exercise and Health, University of Winchester, Winchester, United Kingdom; ^3^Department of Emergency Medicine, School of Medicine, University of North Carolina at Chapel Hill, Chapel Hill, NC, United States; ^4^Department of Exercise and Sport Science, University of North Carolina at Chapel Hill, Chapel Hill, NC, United States; ^5^Department of Biology, Ave Maria University, Ave Maria, FL, United States; ^6^School of Health Sciences, University of Southampton, Southampton, United Kingdom

**Keywords:** arterial stiffness, measurement, vascular risk, Doppler ultrasound, pulse-transit time

## Abstract

**Background:** Carotid-femoral pulse-wave velocity (cfPWV) is the reference standard measure of central arterial stiffness. However, it requires assessment of the carotid artery, which is technically challenging, and subject-level factors, including carotid artery plaque, may confound measurements. A promising alternative that overcomes these limitations is heart-femoral PWV (hfPWV), but it is not known to what extent changes in cfPWV and hfPWV are associated.

**Objectives:** To determine, (1) the strength of the association between hfPWV and cfPWV; and (2) whether change in hfPWV is associated with change in cfPWV when central arterial stiffness is perturbed.

**Methods:** Twenty young, healthy adults [24.0 (SD: 3.1) years, 45% female] were recruited. hfPWV and cfPWV were determined using Doppler ultrasound at baseline and following a mechanical perturbation in arterial stiffness (120 mmHg thigh occlusion). Agreement between the two measurements was determined using mixed-effects regression models and Bland-Altman analysis.

**Results:** There was, (1) strong (ICC > 0.7) agreement between hfPWV and cfPWV (ICC = 0.82, 95%CI: 0.69, 0.90), and, (2) very strong (ICC > 0.9) agreement between change in hfPWV and cfPWV (ICC = 0.92, 95%CI: 0.86, 0.96). cfPWV was significantly greater than hfPWV at baseline and during thigh occlusion (*both P* < 0.001). Inspection of the Bland-Altman plot, comparing cfPWV and corrected hfPWV, revealed no measurement magnitude bias.

**Discussion:** The current findings indicate that hfPWV and cfPWV are strongly associated, and that change in cfPWV is very strongly associated with change in hfPWV. hfPWV may be a simple alternative to cfPWV in the identification of cardiovascular risk in clinical and epidemiological settings.

## Introduction

Pulse wave velocity (PWV) is the gold standard assessment of arterial stiffness and is widely used in epidemiological research to estimate cardiovascular disease (CVD) risk (1). Carotid-femoral PWV (cfPWV), a measure of aortic stiffness, is the reference standard, being a strong independent predictor of CVD risk in both general (2, 3) and patient populations (4). Assessments of cfPWV can be performed with accuracy and precision using tonometric (5), oscillometric (6, 7) or Doppler ultrasound technologies (8–10). However, regardless of the approach, applanation or imaging of the carotid artery is required. This can be technically challenging in certain populations, including persons who are obese and those with advanced carotid artery atherosclerosis (11) and this likely limits its clinical use (12). Also, the carotid artery is not consistent with the path of blood flow for the region of interest - the aortic-illiac pathway. As such it is assumed that the timing of the heart to carotid pressure wave is the same as that from the heart to the descending aorta when calculating cfPWV (13). Finally, cfPWV does not encompass the proximal aorta (14, 15), the distensibility of which is a predictor of CVD risk and mortality (16). One promising alternative measure of central arterial stiffness that overcomes these limitations is heart-femoral PWV (hfPWV).

PWV is calculated by measuring the pulse transit time (PTT) for the arterial waveform to pass between two points of a measured distance. For, hfPWV, PTT can be calculated as the time between the R wave of an electrocardiogram (ECG) and the foot of the femoral pressure waveform. The hfPWV approach confers a number of potential advantages over cfPWV: (i) it is simpler to conduct, as the measurement is not dependent on assessment of the carotid artery; (ii) the measurement path is consistent with the blood flow path; and, (iii) it incorporates the proximal aorta. Although measures of hfPWV have previously been used as a clinical or epidemiological cardiovascular monitoring tool (17–22), only two studies have determined its association with the criterion, cfPWV (13, 23). Both studies reported strong correlations (*r* = 0.81–0.83) between hfPWV and cfPWV in community dwelling healthy middle-aged (13) and older adults (23). However, what has not been determined is to what extent change in hfPWV corresponds to change in cfPWV when central arterial stiffness is perturbed. Whilst hfPWV is valid, to be of utility in clinical or epidemiological environments it must also be demonstrated to accurately track changes in central arterial stiffness and therefore CVD risk.

Central arterial stiffness can be artificially manipulated, including through the use of exercise (24), meal consumption (25) or pharmacological means (26). But these interventions have systemic affects and induce changes in heart rate (HR) which confound cfPWV (27, 28). A simple, non-invasive model that minimizes systemic hemodynamic changes and has been used by others (29) is the inflation of a tourniquet to a low-moderate pressure distal to the arterial segment of interest. This external compression obstructs forward pressure wave propagation, modifies pulse-wave reflection morphology, and alters blood flow profiles, distorting arterial stiffness in upstream vessels (29, 30). This model can be used to confirm whether hfPWV and cfPWV are able to similarly track changes in central arterial stiffness. The aims of this study were to determine: (1) the strength of the association between cfPWV and hfPWV; and (2) whether change (baseline *vs*. cuff inflation) in cfPWV is associated with change in hfPWV.

## Methodology

This study is reported in accordance with CONSORT (Consolidated Standards of Reporting Trials) guidelines (31). Ethical approval was obtained from the University of North Carolina at Chapel Hill Institutional Review Board (17-0745), and all participants provided written informed consent prior to participating in the study.

### Participants

To identify the upper limit for agreement between test and criterion measures, a homogeneous cohort of young (18–40 years of age) healthy adults were recruited to participate in the study. Participants were excluded if they reported any known cardiometabolic disorders, were taking medications known to affect cardiovascular function or reported cigarette smoking. To account for potential influences of hormonal status on study outcomes, premenopausal women were studied during the early follicular phase of their menstrual cycle or during the placebo phase of oral contraceptive use.

### Experimental Design

A single-visit, two condition design, in which cfPWV and hfPWV were determined at rest and during thigh cuff inflation. The order of conditions was not randomized to prevent the impact of cuff inflation on baseline measures. However, the analyst was blinded to the order of testing. After familiarization, participants were tested in an environmentally controlled room (temperature, 22 ± 1°C; relative humidity, 51 ± 2%). All participants had fasted for 12 h and were asked to avoid strenuous physical activity, caffeine and alcohol for 24 h before measurements. After a 20-min rest period in a supine position, right sided cfPWV and hfPWV were determined using Doppler ultrasound. A rapid inflation cuff (SC10; Hokanson Inc., Bellevue, WA, USA), placed immediately proximal to the right knee, was then inflated to a pressure of 120 mmHg. Following a 5-min stabilization period all assessments were repeated. Pilot study [*n* = 5, 31.0 (SD: 4.4) years, 20% female] findings revealed that external compression of 120 mmHg permitted the greatest impact on lower-extremity arterial blood flow, without full occlusion. Ultrasound was used to image the right posterior tibial artery to ensure blood flow was maintained. In each condition, oscillometric pressure waveforms were recorded on the left upper arm, from which central haemodynmic measurements were derived. Stroke volume (SV), cardiac output (CO) and total peripheral resistance (TPR) were also determined.

### Experimental Measures

#### Pulse Wave Velocity

A LOGIQ P6 ultrasound device equipped with an 11–2 mHz linear array probe (GE Healthcare, Wauwatosa, USA) was used to sequentially scan and obtain ECG-gated pulse-wave Doppler waveforms at the right common carotid artery (CCA) and right superficial femoral artery (SFA) by a single trained operator. The CCA was imaged 2 cm proximal to the carotid bulb and the SFA was imaged 2 cm distal to the bifurcation from the common femoral artery. The US beam was placed at mid-vessel and was angled at ≤60° relative to the longitudinal axis of the artery to permit simultaneous measurement of brachial diameter and blood velocity. An effort was made to ensure that the vessel clearly extended across the entire (un-zoomed) imaging plane to minimize the risk of skewing the vessel walls. Ultrasound global (acoustic output, gain, dynamic range, gamma and rejection) and probe-dependent (zoom factor, edge enhancement, frame averaging and target frame rate) settings were standardized. Three 10 s video recordings were obtained at each position during which participants were asked to hold their breath to avoid any respiratory noise.

**Figure 1:**
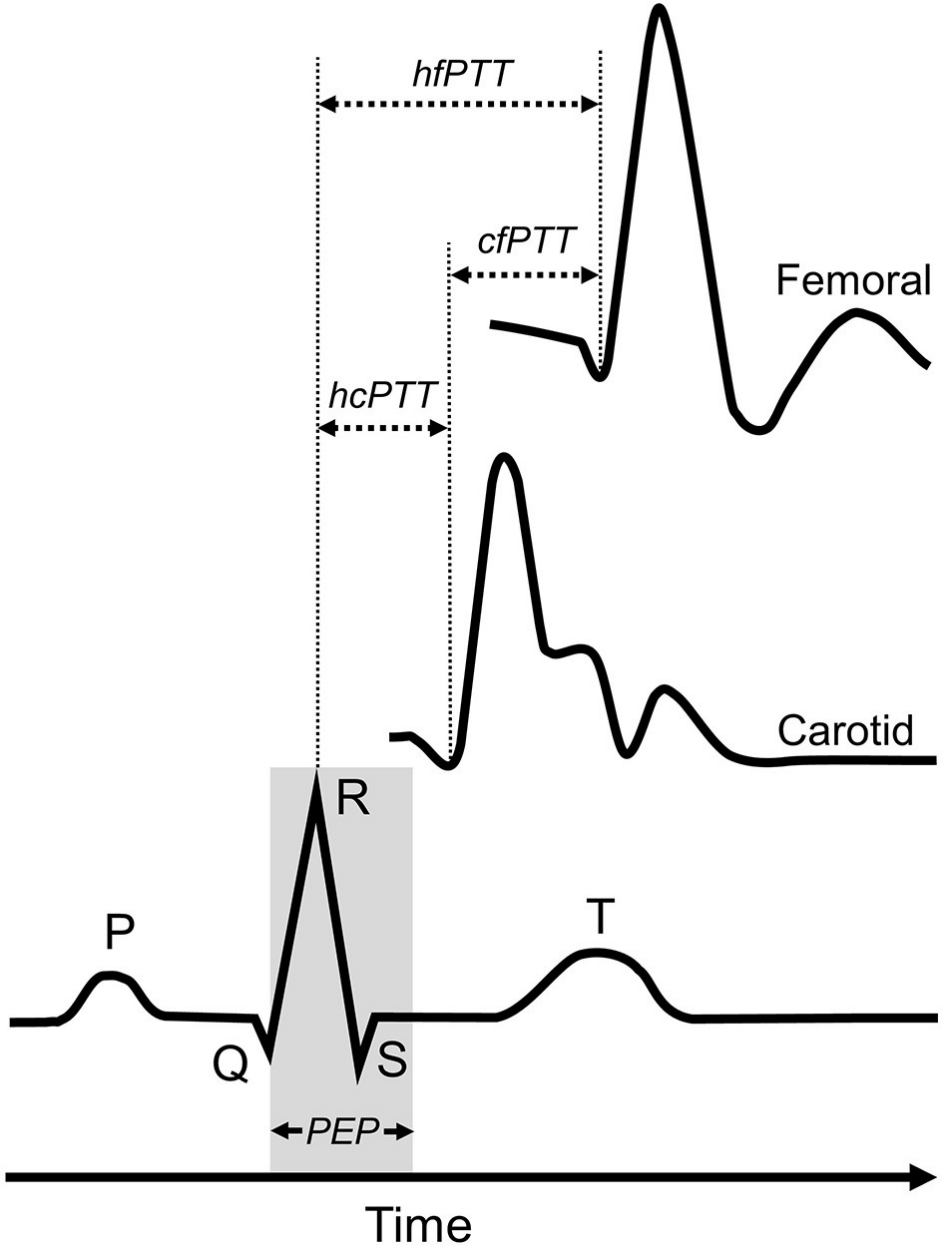
Determination of pulse-transit time (PTT) for heart-femoral pulse-wave velocity and carotid-femoral pulse-weave velocity measures. Carotid-femoral PTT (cfPTT) was calculated as heart-femoral PPT (hfPTT) minus heart-carotid PTT (cfPTT). PEP, pre-ejection period.

An image was obtained from the beginning, middle and end of each video (i.e., 3 images × 3 videos for each measurement site). Images were analyzed offline using ImageJ (Version 1.51q, National Institutes of Health, Bethesda, USA) (32) by a single blinded analyst. The interval between the r-wave of the QRS complex and the foot of the systolic upstroke in the Doppler spectral envelope was measured and averaged over at least three consecutive cardiac cycles for each image at carotid and femoral sites (8, 33). The foot of the Doppler waveform was identified by the intersection of the upstroke of the wave with the baseline of zero frequency. ECG gated pulse-wave Doppler waveforms at both carotid and femoral arterial sites were used to determine carotid-femoral PTT (cfPTT), which represented the interval of time between the foot of the carotid and femoral arterial waveforms and calculated as heart-femoral PTT minus heart-carotid PTT (Figure 1). The cfPWV arterial path length (*D*) was estimated by measuring the linear distance from the suprasternal notch to the mid-point of the probe at the SFA and subtracting the distance from the suprasternal notch to the mid-point of the probe at the CCA. Path length was directly measured, using a custom device to bypass body contours. The r-wave to the foot of the femoral Doppler waveform interval represented the heart-femoral PTT (hfPTT) and estimated *D* was the linear distance from the suprasternal notch to the mid-point of the probe at the SFA only. Pulse-wave velocity was calculated as: PWV = *D*/PTT.

#### Mechanistic Explanatory Variables

It is recognized that HR, mean arterial pressure (MAP) and arterial wave reflection morphology can impact central arterial stiffness. Accordingly, in order to determine the systemic effects of cuff inflation and their potential contribution to changes in central arterial stiffness, (i) central pressure and indices of wave reflection were determined using pulse wave analysis (PWA), and, (ii) CO, SV and TPR were determined using plethysmography. For PWA, oscillometric pressure waveforms were recorded on the left upper arm by a single observer using the SphygmoCor XCEL device (AtCor Medical, Sydney, Austrailia), following standard manufacturer guidelines (34). Each single measurement cycle consisted of a 60 s brachial blood pressure recording followed by a 10 s sub-systolic recording. A corresponding aortic pressure waveform was then generated using a validated transfer function (35), from which central systolic blood pressure (cSBP), augmentation index (AIx), augmentation pressure (AP), forward aortic pressure (Pf) and backward aortic pressure (Pb) were derived. Beat-to-beat CO, SV and TPR were monitored non-invasively at the finger (Human NIBP Nano System, Finapres Medical Systems B.V., Netherlands) and sampled at 200 Hz via PowerLab systems (LabChart 8, ADIinstruments, Australia). Presented data represents the mean of three 1-min periods at the start, middle and end of ultrasound data collection.

### Sample Size

Sample size estimates were conducted using G^*^Power 3. For calculating the association between measures, we elected to use a mixed model to increase statistical power by enabling repeated measures to be nested within each subject. Using this approach, a sample of 37 data points can detect a 1-tailed correlation of 0.4 (moderate) with 80% power and a 5% chance of a type 1 error. For a mixed model-based approach to correlation, the degrees of freedom are equal to *N* – 1, where *N* is the total number of participants (36). For a sample size of 20, with two repeated measures, the degrees of freedom (number of required participants) is 40 – 1 = 39.

### Statistical Analysis

Statistical analyses were performed using RKWard (2019, Version 0.7.1), a frontend to the R statistical package. The significance level was set a *priori* for all statistical procedures at α = 0.05. Raw data are presented as mean [standard deviation (SD)] and mixed model data are presented as mean [95% confidence interval (95%CI)]. The corresponding author (KS) had full access to the data in the study and was responsible for the integrity of the data set and the data analysis.

Paired *t-*tests were used to compare baseline and cuff inflation hemodynamic responses, as well as measures of cfPWV and hfPWV within conditions. Effect sizes (ES) were calculated as Cohen's *d* where <0.2 was defined as trivial, 0.2–0.3 as small, 0.4–0.8 as moderate, and >0.8 as large. The association between the criterion and test measurements was determined using nested mixed-effects regression models. This approach allowed us to maximize statistical power while accounting for the correlated error variances (repeated measures) and the condition (base, cuff) structure. Specifically, the test measure was regressed against the criterion measure and nested within subject and condition (base, cuff inflation). Subject and cuff intercepts were specified as random effects and used to estimate the between-subject (σ2s), between-condition (σ2c) and residual (σ2r) variance. Subsequently, the between-measurement ICC was calculated as σ2s/(σ2s + σ2r) and the between condition ICC as σ2s/(σ2c + σ2r). To adjudicate the strength of association we used existing criteria for Pearson product-moment correlation (*r*). Although there is no universal criterion, in general, r value estimates of <0.2, 0.2–0.4, 0.4–0.7, 0.7–0.9, and >0.9 indicate negligible, weak, moderate, strong, and very strong agreement, respectively (37). To ensure transparency in our statistical approach, the supplement also reports associations between measurements for each condition using the standard Pearson product moment correlation, and between measurement correlations rmcorr package for R. As hfPWV was found to be on a different scale to cfPWV, to permit direct comparison and visual analysis of the uniformity of error over the range of participant measurement values (38), hfPWV was corrected (hfPWVc) using the mixed model regression equation and subsequently used to generate Bland–Altman plots with hfPWVc and cfPWV on comparable scales (38).

## Results

All participants were included in analyses and there was no missing participant data. Participant descriptive characteristics are presented in Table 1.

**Table 1:** Descriptive characteristics of study participants.

***n* = 20**	**Mean**	**(SD)**
Age (years)	24	(3.1)
Female (%)	45	
Height (m)	1.7	(7.3)
Weight (kg)	70.2	(11.4)
BMI (kg/m^2^)	23.9	(2.5)
PA (avg/mins/wk)	65.0	(21.1)
PA Sessions (avg/wk)	4.6	(1.4)

*BMI, body mass index; PA, physical activity*.

### Blood Pressure and PWV Response to Cuff Inflation

Blood pressure and PWV responses to cuff inflation are reported in Table 2. None of the peripheral or central haemodynamic measures were significantly impacted by cuff inflation (all *P* >0.05). There was large effect (ES > 0.8) decrease in hfPWV (−0.10 m/s, 95%CI: −0.07, −0.14) and a moderate effect (ES 0.4–0.8) decrease in cfPWV (−0.22 m/s, 95%CI: −0.06, −0.38) with cuff inflation.

**Table 2:** Blood pressure, wave reflection, carotid-femoral pulse wave velocity (cfPWV) and heart-femoral pulse wave velocity (hfPWV) responses to cuff inflation (*n* = 20).

	**SBP**	**DBP**	**MAP**	**HR**	**cSBP**	**AP**	**AIx**	**Pb**	**Pf**	**SV**	**CO**	**TPR**	**hfPWV**	**cfPWV**
	**(mmHg)**	**(mmHg)**	**(mmHg)**	**(beats min-1)**	**(mmHg)**	**(mmHg)**	**(%)**	**(mmHg)**	**(mmHg)**	**(ml)**	**(l min-1)**	**(mmHg.s)**	**(m/s)**	**(m/s)**
**Mean**														
Rest	117	69	81	55	102	1	3	12	26	57	3.3	4.0	2.97	4.87
120 mmHg	118	70	81	57	102	0	1	13	26	54	3.2	3.8	2.87	4.65
**Standard Deviation**														
Rest	12	5	6	9	10	3	10	3	5	10	0.6	1.0	0.24	0.45
120 mmHg	13	6	9	10	11	7	10	5	5	11	0.6	0.7	0.20	0.45
**Condition Effect**														
β	1.28	1.25	0.15	1.35	0.70	−1.55	−2.00	0.80	−0.40	−2.80	−0.12	−0.29	−0.10	−0.22
P	0.747	0.494	0.954	0.659	0.827	0.382	0.552	0.556	0.812	0.393	0.562	0.287	<0.001	0.013
ES	0.05	0.11	0.01	0.07	0.03	−0.14	−0.09	0.09	−0.04	−0.14	−0.09	−0.17	−0.84	−0.43

*MAP, mean arterial pressure; HR, heart rate; cSBP, central systolic blood pressure; AP, aortic pressure; AIx, augmentation index; Pb, aortic backward wave pressure; Pf, aortic forward wave pressure; SV, stroke volume; CO, cardiac output; TPR, total peripheral resistance; ES, Cohen's d effect size*.

### Measurement Comparison

**Figure 2:**
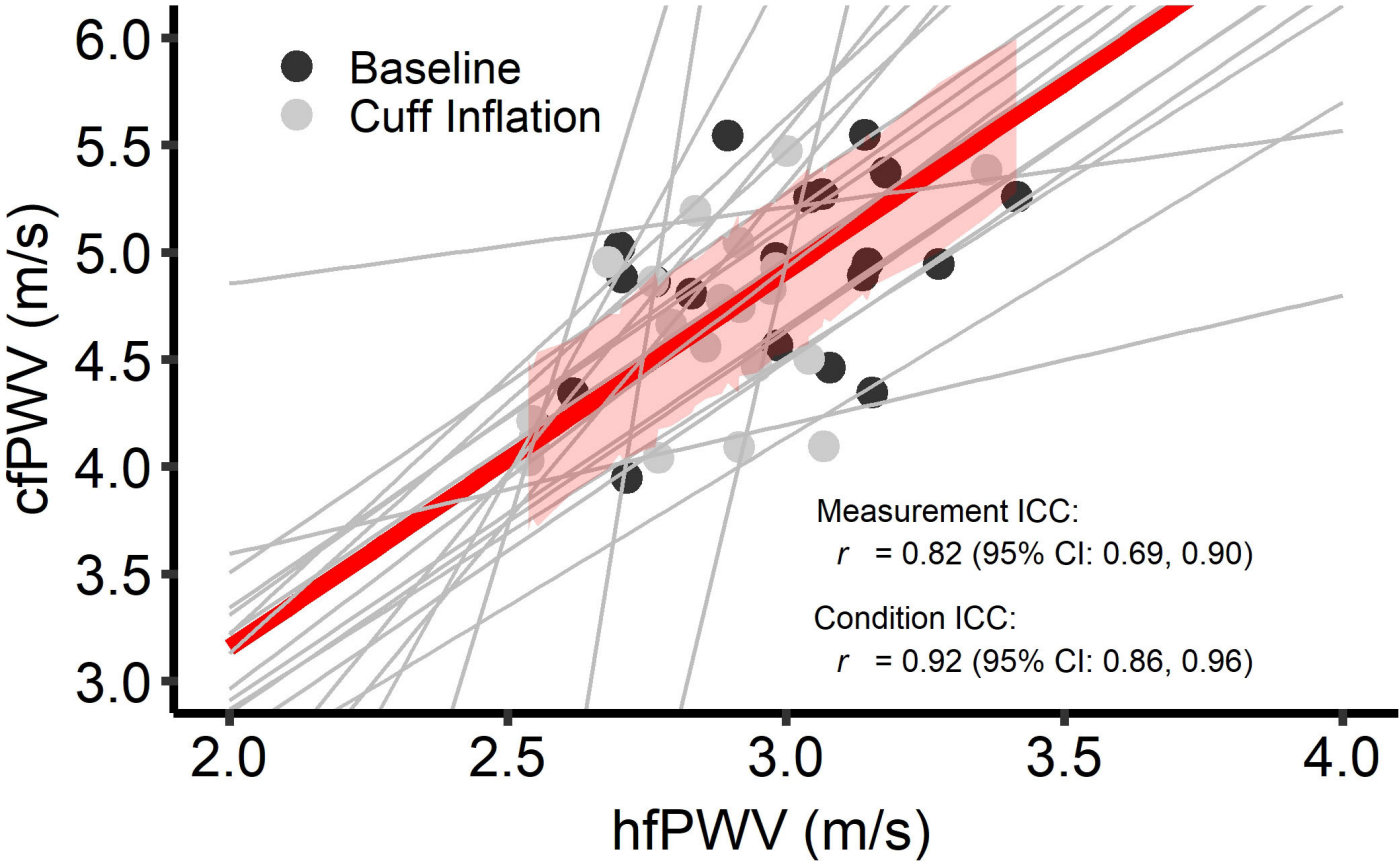
Intra-class correlations (ICC) for between-measurement (i.e., overall comparison between criterion and test measure) and between-condition [i.e., change (cuff – base) in the test measure *vs*. change in the criterion measure] carotid-femoral pulse wave velocity (cfPWV) and heart-femoral pulse wave velocity (hfPWV) comparisons. *n* = 20. Red line and red shading depict overall between-measurement agreement and standard error, respectively. Gray lines depict between-condition agreement.

**Figure 3:**
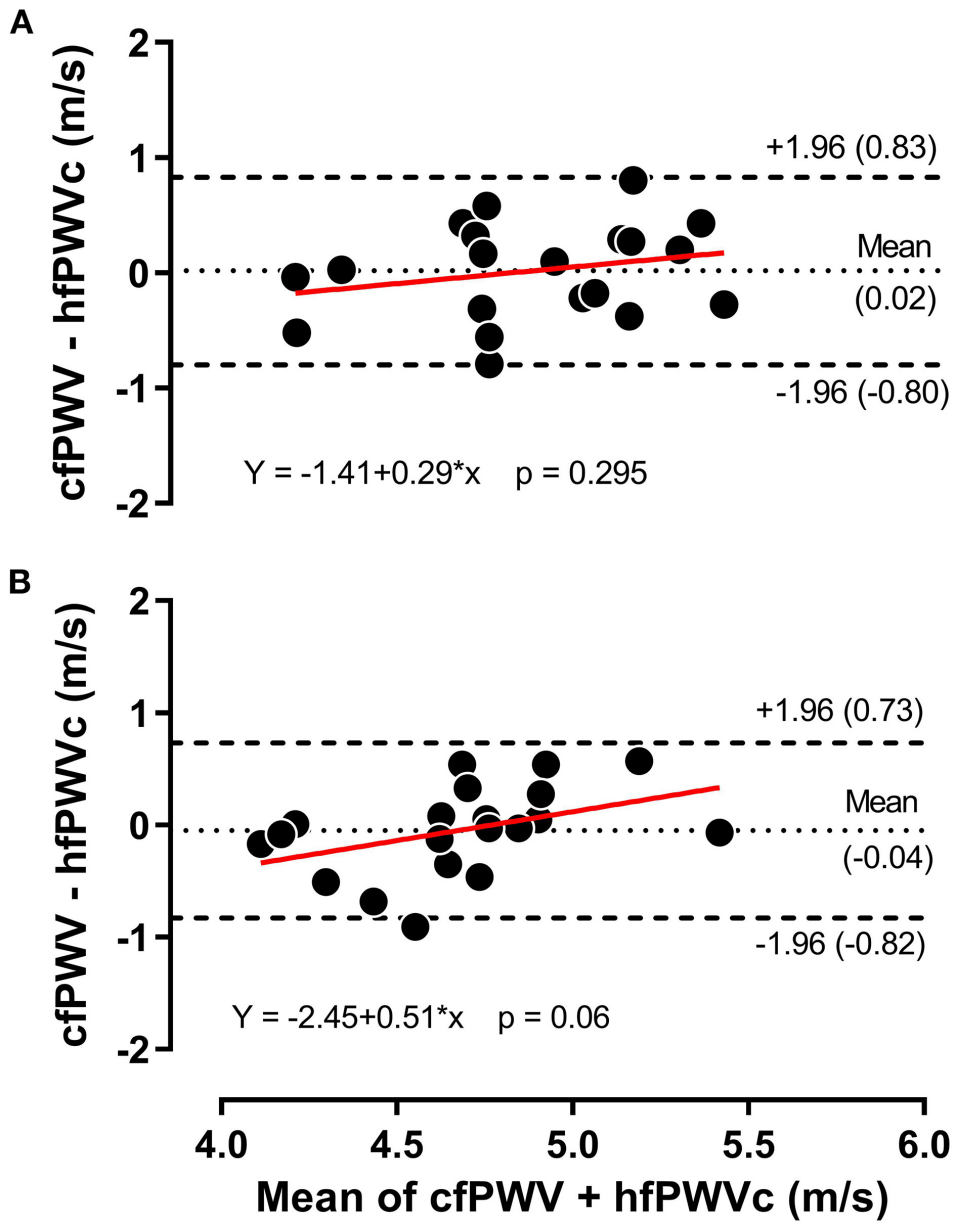
Bland-Altman plot for carotid-femoral pulse wave velocity (cfPWV) vs. corrected heart-femoral pulse wave velocity (hfPWVc) at baseline **(A)** and during 120 mmHg thigh cuff inflation **(B)**, *n* = 20.

Figure 2 presents the comparison between hfPWV and cfPWV. There was strong between-measurement agreement (ICC: 0.82, 95%CI: 0.69–0.90) and very strong between-condition agreement (ICC: 0.92, 95%CI: 0.86–0.96) for hfPWV and cfPWV comparison. Measures of cfPWV were significantly higher than hfPWV at baseline (mean difference: 1.9 m/s, 95%CI: 1.72–2.09, *P* < 0.001) and cuff inflation (mean difference: 1.78 m/s, 95%CI: 1.6–1.97, *P* < 0.001). Mean bias for Bland-Altman analysis comparing cfPWV and hfPWVc was 0.02 m/s (95%CI: −0.18 to 0.21) at baseline and −0.04 m/s (95%CI: −0.24 to 0.14) at cuff inflation. Inspection of the plots indicated no measurement magnitude bias at baseline or cuff inflation (Figures 3A,B).

### Ancillary Analysis

Ancillary analysis is provided in the supplement. Pulse transit time and arterial path length values for the determination of cfPWV and hfPWV are presented in Supplementary Table 1. To ensure full transparency, Bland-Altman plots generated using uncorrected hfPWV and cfPWV are presented in Supplementary Figure 1. The associations between measurements for each condition are reported using standard Pearson product moment correlations in Supplementary Figure 2. To further interrogate intra-individual associations between change (cuff vs. baseline) in cfPWV and change in hfPWV repeated measures correlation was determined (Supplementary Figure 3).

## Discussion

This study investigated, (1) the strength of the association between cfPWV and hfPWV; and (2) whether change in cfPWV is associated with change in hfPWV. Our main findings show that there is a strong (ICC > 0.7) agreement between hfPWV and cfPWV, and that change (baseline vs. cuff inflation) in hfPWV and change in cfPWV demonstrated very strong agreement (ICC > 0.9).

### Limitations and Strengths

The limitations and strengths of this study are addressed to best contextualize the findings. Firstly, a relatively homogeneous group of young, healthy participants were recruited to ascertain the upper limit of agreement between changes in cfPWV and hfPWV measures, thus limiting the overall generalizability of our findings to populations of varying age and health states. Prior to clinical use, it is imperative to identify whether any inherent error, bias or variability is caused by the technique itself and not a consequence cardiovascular pathology. A major strength is that this is the first study to directly compare changes in the reference standard measure of central arterial stiffness, cfPWV, and a novel, simpler alternative hfPWV.

### Comparison to Literature

In the only other studies to directly compare cfPWV and hfPWV measures (13, 23), comparable associations (*r* = 0.81–0.83) were observed to that reported in the present study (ICC = 0.82). However, in contrast to Stoner et al. (23) who reported significant bias at higher PWVs, we observed no measurement magnitude bias between cfPWV and corrected hfPWV measures. This contrast may be due to the inclusion of young, healthy individuals in the present study, whereas Stoner et al. (23), studied a large population of older adults of varying health and disease states. Higher PWV values in older adults may be accompanied by greater carotid plaque prevalence, which could influence local arterial mechanics and vessel elasticity (39), potentially leading to greater error variance. Unlike cfPWV, hfPWV measures do not require carotid artery assessment and are therefore not confounded by the presence of plaque.

The present study also extends previous findings by being the first to report that following a perturbation to acutely manipulate central arterial stiffness, changes in cfPWV and hfPWV also demonstrate very strong agreement (ICC = 0.92). In the intended absence of systemic changes, the reduction in central arterial stiffness is likely a consequence of lower limb blood pooling, induced by venous outflow occlusion, leading to a dampening of wave reflection and an attenuation of central pressure (40). Of note, Stoner et al. (23) did report that sex impacted the agreement between hfPWV and cfPWV; with a superior agreement in women, but a greater error variance at higher PWV's. It is recognized that vascular structural and functional properties differ between sexes (41, 42) and there are divergent relationships between HR and cfPWV (43). Accordingly, whilst the present study was not adequately powered to examine sex differences, future studies should identify if the agreement between changes in cfPWV and hfPWV is impacted by sex.

Measures of cfPWV were significantly higher than hfPWV measures; a difference that may have been driven by several sources. Firstly, there is an intrinsic difference between hfPWV and cfPWV in the start point from which PTT is measured (see Figure 1). cfPTT is determined as the interval between the foot of the upstroke of carotid and femoral waveforms. Hence, PTT is inversely proportional to PWV, being influenced by the same arterial and haemodynamic factors. In contrast, hfPTT, or pulse arrival time, is determined as the interval between the r-wave of the QRS complex and the foot of the upstroke of the femoral waveform (8, 33). As such, hfPTT includes the pre-ejection period (PEP), which is the electromechanical delay associated with the conversion of electrical signal into mechanical pumping force and isovolumetric contraction to open the aortic valves (44, 45). hfPWV may therefore not represent a true PWV, consequently overestimating aortic PTT. One potential solution is to measure PTT from the beginning of the S segment of the ECG, thereby reducing the impact of PEP on hfPWV measures. However, in comparison to the prominent R-wave, the S-wave is susceptible to motion and measurement artifact and is likely less reliable. Alternatively, isovolumetric contraction time, and thus PEP, can be estimated by analyzing the heart to carotid waveform and using cfPWV as a surrogate for ascending aorta PWV (46). But whilst this promising deductive approach demonstrated good agreement with echocardiography derived PEP, a number of assumptions are made, including; (i) ascending aortic length, and (ii) that the electro mechanical delay of PEP is constant and equal to the QR interval (46). These assumptions may not always be appropriate, particularly within patient populations. Secondly, cfPWV measures do not encompass the proximal aorta (13–15). cfPWV assessment employs the carotid artery, which is not consistent with the path of blood flow for the region of interest - the aortic-illiac pathway. To adjust for this, the arterial segment from the ascending aorta to the carotid artery is omitted (subtracted) from the effective path lengths used for cfPWV calculations (14). In doing so, it is also assumed that the time the forward pressure wave takes to travel from the heart to the carotid artery is the same time it takes a simultaneous wave to travel the same distance from the heart to the descending aorta (13, 14). Therefore, cfPWV actually represents the PWV from the descending aorta to the femoral artery. Omission of the highly elastic proximal aorta inherently leads to cfPWV measures being higher than hfPWV measures, as the latter includes the whole aorta. Hickson et al. (47) demonstrated that exclusion of the aortic arch from global aortic PWV measures determined using magnetic resonance imaging reduced the difference between (up to 0.8 m/s) and improved the agreement with cfPWV determined using both tonometric and oscillometric technologies. Although the inclusion of PEP and the ascending aorta collectively contribute to causing greater divergence, hfPWV and cfPWV are consistently proportionate to each other, hence their strong agreement, as reported in the present study and by others (13, 23).

### Implications

Despite cfPWV being a strong independent predictor of CVD risk (2–4), it is not widely used in clinical practice. Technical challenges associated with the assessment of the carotid artery, which can be difficult to perform in certain populations, likely limits it clinical use. Furthermore, although not widely recognized, the inherent omission of the proximal aorta may overlook important pathophysiological information (16). hfPWV is a simpler alternative which is less likely to be confounded by subject-level factors, including the presence of carotid plaque, and encompasses the predictive capacity of the whole aorta. Previously cfPWV and hfPWV measurements have demonstrated good baseline agreement. The current study extends the scant hfPWV literature by reporting that cfPWV and hfPWV demonstrate strong agreement when tracking acute changes in central arterial stiffness.

Measures of hfPWV are inherently lower than cfPWV. It may be possible to correct hfPWV to ensure greater comparability with cfPWV, however such corrections may limit measurement precision. Although there is some divergence in absolute PWV values, hfPWV and cfPWV are proportionate to one another. It is recognized that hfPWV measures still require assessment of the femoral artery. Like the carotid artery, femoral artery assessments can also be challenging and may require a highly trained operator to ensure precision, particularly when using ultrasound or tonometry technologies. One emerging technique which is operator independent and has demonstrated moderate agreement with cfPWV (*r* = 0.64) is the determination of hfPWV using an oscillometric thigh cuff to detect femoral waveforms (22). Further exploration of this technology is required, but the simplicity of the oscillometric-based hfPWV measures may encourage the clinical adoption of central arterial stiffness phenotyping as a marker of arterial aging and CVD risk. Regardless of approach, future work should seek to identify the repeatability of hfPWV measures and if hfPWV and cfPWV similarly track central arterial stiffness changes in older adults with CVD progression.

## Conclusions

The current findings indicate that cfPWV and hfPWV are strongly associated, and that change in cfPWV is very strongly associated with change in hfPWV following the acute perturbation of central arterial stiffness. Accordingly, hfPWV may be a simple, viable alternative to cfPWV in the tracking of CVD risk within clinical and epidemiological environments.

## Data Availability Statement

The raw data supporting the conclusions of this article will be made available by the authors, without undue reservation.

## Ethics Statement

The studies involving human participants were reviewed and approved by the University of North Carolina at Chapel Hill Office of Human Research Ethics (17-0745). The patients/participants provided their written informed consent to participate in this study.

## Author Contributions

KS, SF, JF, and LS contributed to the conception and design of the experiment, data collection, analysis, interpretation of the data, and the drafting of the manuscript. GZ, CP, KB, and EK contributed to data collection and drafting of the manuscript. MM, DC, and DL contributed to data interpretation and the drafting of the manuscript. All authors approved the final version of the manuscript and agree to be accountable for all aspects of the work in ensuring that questions related to the accuracy or integrity of any part of the work are appropriately investigated and resolved and persons designated as authors qualify for authorship, and all those who qualify for authorship are listed.

## Conflict of Interest

The authors declare that the research was conducted in the absence of any commercial or financial relationships that could be construed as a potential conflict of interest.
