# Supplementary material for: Acute Changes in Carotid-Femoral Pulse-Wave Velocity Are Tracked by Heart-Femoral Pulse-Wave Velocity
Source: Front Cardiovasc Med. 2021 Jan 20;7:592834. doi: 10.3389/fcvm.2020.592834 (PMC7854542; doi:10.3389/fcvm.2020.592834)
Supplement: Supplementary file 1 [file Data_Sheet_1.pdf]

SUPPLEMENTAL MATERIAL

Table S1: Arterial path length (D) and pulse-transit time (PTT) values for the calculation of carotid-femoral (cf-) and heart-femoral (hf-) pulse-wave velocity (PWV) variables.

n=20	hfD (mm)	hfPTT (ms)	hfPWV (m/s)	cfD (mm)	cfPTT (ms)	cfPWV (m/s)
**Mean**						
Rest	599	203	2.97	513	106	4.87
120 mmHg	-	210	2.87	-	110	4.65
**Standard Deviation**						
Rest	37	15	0.24	43	10	0.45
120 mmHg	-	14	0.20	-	8	0.45

FIGURE S1. Bland-Altman plot for carotid-femoral pulse wave velocity (cfPWV) versus uncorrected heart-femoral pulse wave velocity (hfPWVc) at baseline (A) and during 120mmHg thigh cuff inflation (B), n = 20. Dotted line denotes mean bias. Dashed lines represent bias upper and lower 95% confidence intervals. Solid black line depicts overall linear regression.

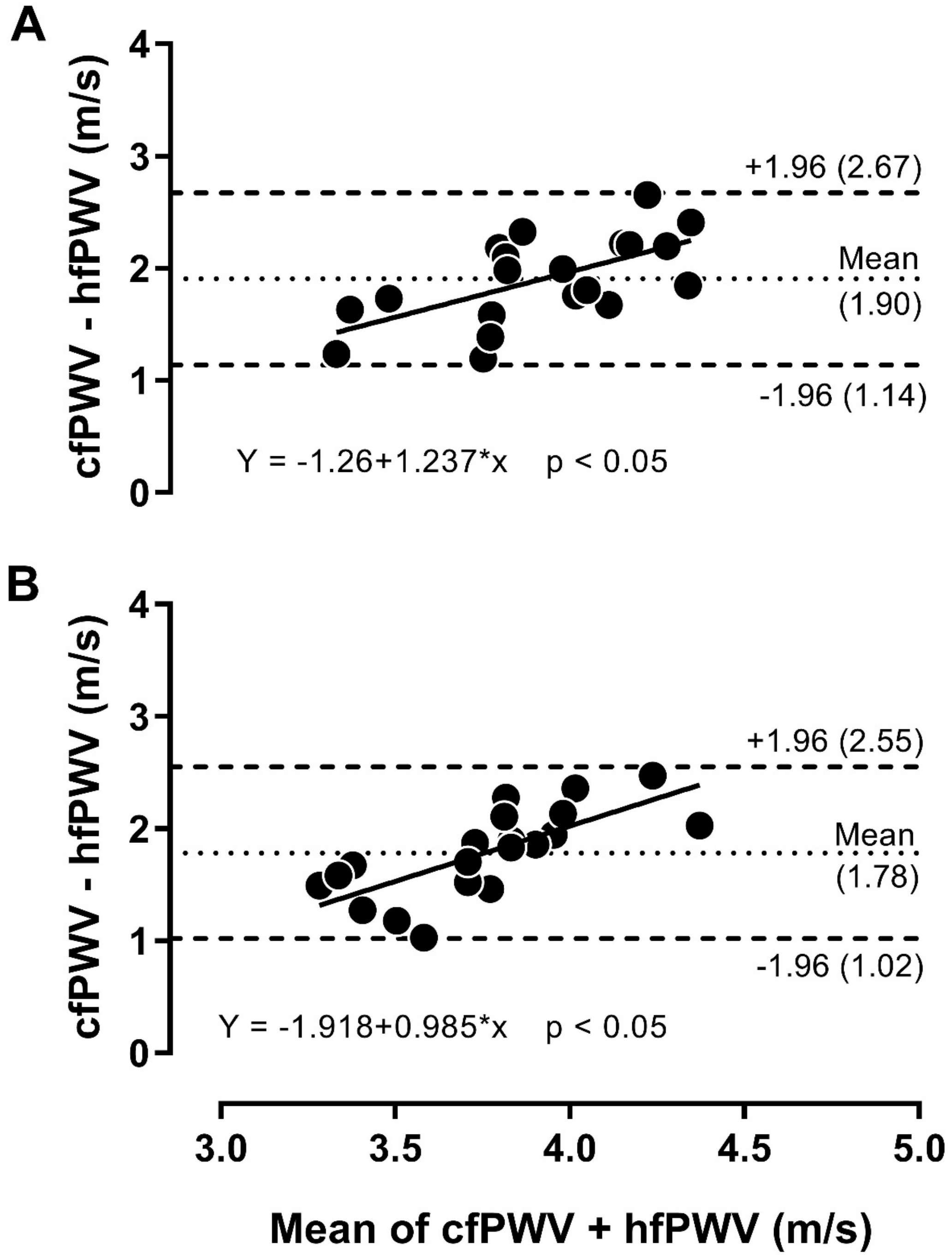

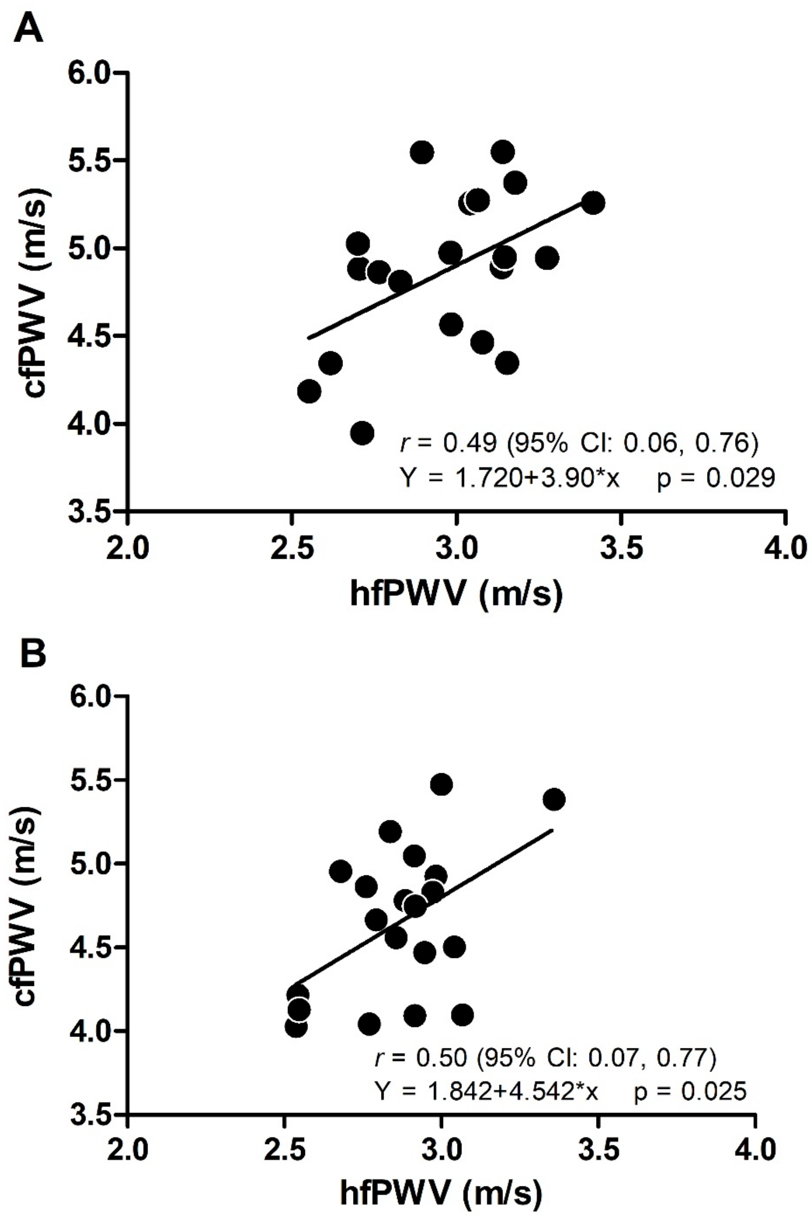

Figure S2: Correlations between carotid-femoral pulse wave velocity (cfPWV) and heart-femoral pulse wave velocity (hfPWV) at rest (A) and during 120 mmHg cuff inflation (B), n=20. **Interpretation**: There were significant moderate (r = 0.4-0.7) positive correlations between hfPWV and cfPWV at baseline and during cuff occlusion ($P < 0.05$).

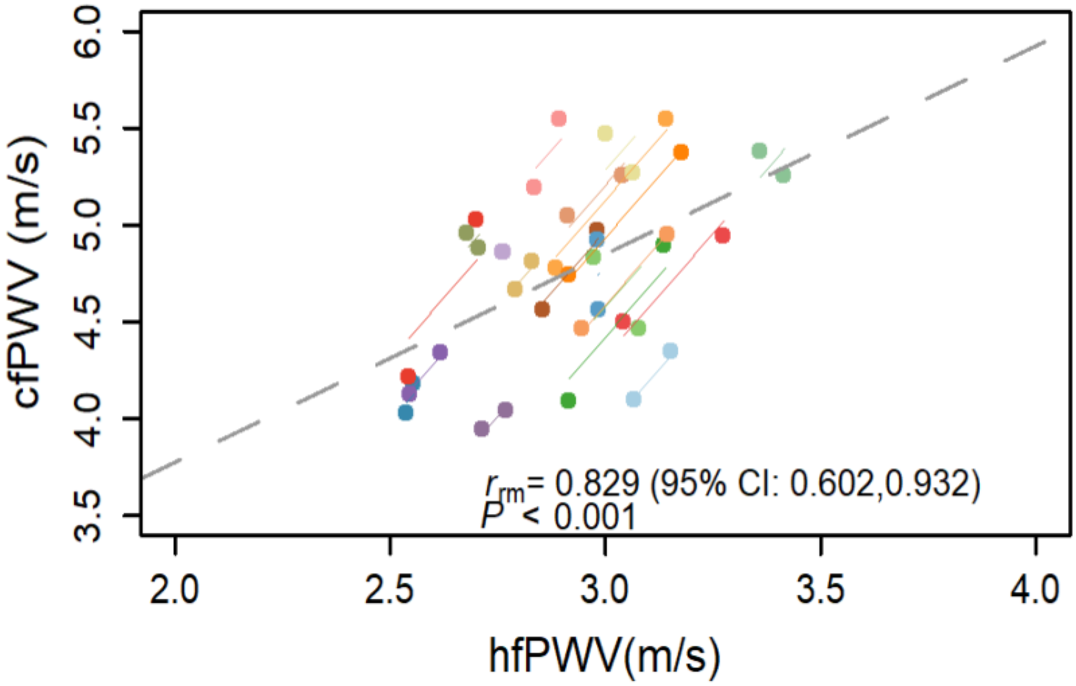

FIGURE S3. Repeated measures correlation analysis between carotid-femoral pulse wave velocity (cfPWV) and heart-femoral pulse wave velocity (hfPWV), n=20. **Interpretation**: There was a significant strong (r = 0.7- 0.9) positive correlation between hfPWV and cfPWV across conditions (*P* < 0.05).
